# Multimorbidity, physical frailty, and self-rated health in older patients with atrial fibrillation

**DOI:** 10.1186/s12877-020-01755-w

**Published:** 2020-09-11

**Authors:** Hawa O. Abu, Jane Saczynski, Jordy Mehawej, Tenes Paul, Hamza Awad, Benita A. Bamgbade, Isabelle C. Pierre-Louis, Mayra Tisminetzky, Catarina I. Kiefe, Robert J. Goldberg, David D. McManus

**Affiliations:** 1 Division of Cardiovascular Medicine, Department of Internal Medicine, University of Massachusetts Medical School, 55 Lake Avenue North, Worcester, MA 01655 USA; 2 Department of Pharmacy and Health Systems Sciences, School of Pharmacy, Northeastern University, Boston, MA USA; 3 Departments of Community Medicine and Internal Medicine, Mercer University School of Medicine, Macon, GA USA; 4 Division of Geriatrics, Department of Medicine, University of Massachusetts Medical School, and Meyers Primary Care Institute, Worcester, MA USA; 5 Department of Population and Quantitative Health Sciences, University of Massachusetts Medical School, Worcester, MA USA

**Keywords:** Geriatric cardiology, Functional status, Well-being, Comorbid diseases

## Abstract

**Background:**

Holistic care models emphasize management of comorbid conditions to improve patient-reported outcomes in treatment of atrial fibrillation (AF). We investigated relations between multimorbidity, physical frailty, and self-rated health (SRH) among older adults with AF.

**Methods:**

Patients (*n* = 1235) with AF aged 65 years and older were recruited from five medical centers in Massachusetts and Georgia between 2015 and 2018. Ten previously diagnosed cardiometabolic and 8 non-cardiometabolic conditions were assessed from medical records. Physical Frailty was assessed with the Cardiovascular Health Study frailty scale. SRH was categorized as either “excellent/very good”, “good”, and “fair/poor”. Separate multivariable ordinal logistic models were used to examine the associations between multimorbidity and SRH, physical frailty and SRH, and multimorbidity and physical frailty.

**Results:**

Overall, 16% of participants rated their health as fair/poor and 14% were frail. Hypertension (90%), dyslipidemia (80%), and heart failure (37%) were the most prevalent cardiometabolic conditions. Arthritis (51%), anemia (31%), and cancer (30%), the most common non-cardiometabolic diseases. After multivariable adjustment, patients with higher multimorbidity were more likely to report poorer health status (Odds Ratio (OR): 2.15 [95% CI: 1.53–3.03], ≥ 8 vs 1–4; OR: 1.37 [95% CI: 1.02–1.83], 5–7 vs 1–4), as did those with more prevalent cardiometabolic and non-cardiometabolic conditions. Patients who were pre-frail (OR: 1.73 [95% CI: 1.30–2.30]) or frail (OR: 6.81 [95% CI: 4.34–10.68]) reported poorer health status. Higher multimorbidity was associated with worse frailty status.

**Conclusions:**

Multimorbidity and physical frailty were common and related to SRH. Our findings suggest that holistic management approaches may influence SRH among older patients with AF.

## Background

Among patients with atrial fibrillation (AF), considerable attention has been paid to long-term clinical outcomes, adherence to, and effectiveness of AF therapy [1, 2]. The onset of AF is often associated with distressing symptoms such as heart palpitations, extreme fatigue, and shortness of breath [3]. Symptoms and fear of complications from AF can lead to significant impairment in patient’s health status, especially in the setting of multimorbidity and accompanying frailty in older patients. Since self-rated health (SRH) status is a major target of therapeutic interventions including catheter and surgical ablation in many patients with AF [4, 5], understanding the factors that are related to SRH are of great importance.

The incidence of AF increases with advancing age, affecting approximately 1 in 10 adults aged 65 years and older in the US [6]. Since AF occurs more commonly in the elderly who have a higher burden of multimorbidity, the combination of underlying AF and coexisting diseases may lead to increasingly impaired functional status [7, 8]. Furthermore, there are complex interactions between a patient’s history of chronic disease and their overall well-being depending on the type and combination of conditions, whether the comorbid conditions interact or have synergistic effects, and the patient’s frailty status [9].

Frailty, a concept distinct from multimorbidity, is a multi-dimensional syndrome characterized by heightened vulnerability to stressors with accompanying low physiological reserve in the presence of multiple organ impairment [10]. Frailty often occurs in the setting of normal aging and can progress in severity with more profound disability [10]. There is a lack of consensus in defining frailty which could range from vulnerability in physical, nutritive, cognitive, or sensory domains to impairment in one’s gait speed or handgrip strength as indicators of frailty [11]. In the present study, we assess physical frailty phenotype developed by Fried and colleagues based on their work using two large epidemiologic studies, the Cardiovascular Health Study and Women’s Health and Aging Studies [12]. With a greater proportion of elderly patients seeking care for AF, frailty should be increasingly considered in clinical decision-making process as a factor that may affect patient’s health status.

The World Health Organization recommends the use of a person’s SRH status as an important indicator of their overall health, a concept that captures aspects of one’s biological, physical, social, and mental functioning [13]. SRH has been linked with a range of health outcomes including hospitalizations, morbidity, and mortality in the general population [14, 15] and in different patient populations [16, 17].

In older patients with AF, there is a need for greater understanding of how the presence of multimorbidity and physical frailty may affect their SRH. The objectives of the present observational study were to describe the overall SRH status of a large cohort of older men and women with AF and examine the independent cross-sectional associations between multimorbidity, physical frailty, and SRH status. We hypothesized that multimorbidity and physical frailty would be prevalent in our older study population and may be associated with poorer functional status and SRH.

## Methods

### Study population

We used baseline data from the prospective multi-center study entitled “Systematic Assessment of Geriatric Elements in AF (SAGE-AF)”. Details of participant recruitment and study protocols have been described previously [18–20]. Participants were recruited from three medical centers in Massachusetts and two medical centers in Central Georgia between 2015 and 2018. Eligible participants were patients aged 65 years and older who had a diagnosis of AF with the presence of arrythmia on electrocardiography tracings, a Holter monitor, or if AF was documented in any clinic or hospital medical record. The Institutional Review Boards at participating sites approved this study. Written informed consent was obtained from each eligible participant prior to formal study enrollment. Data was abstracted from hospital medical records by trained research personnel. Additionally, face-to-face or telephone interviews were conducted with enrolled participants.

### Assessment of multimorbidity

Eighteen previously diagnosed chronic comorbid conditions were assessed from participants’ electronic medical records. These conditions were chosen based on the comorbidities identified and recommended for multimorbidity research by the US Department of Health and Human Services (US-DHHS) Strategic Framework on Multiple Chronic Conditions [21]. Among the US-DHHS list of 20 conditions, we excluded those conditions with a very low prevalence in our study population or were not readily available in the medical records including autism (*n* = 0), human immunodeficiency viral infection (HIV; *n* = 0), schizophrenia (*n* = 0), illicit drug use (*n* = 11), and liver disease (*n* = 31). Patients with dementia were not eligible for study enrollment to avoid undue burden from study interviews. A diagnosis of osteoporosis was not ascertained from medical records due to inconsistency/incomplete documentation. For purposes of understanding the impact of varying chronic conditions on patients’ SRH status, we classified the 18 conditions as either cardiometabolic or non-cardiometabolic. Ten cardiometabolic conditions were assessed including hypertension, dyslipidemia, diabetes, heart failure, valvular heart disease, cardiomyopathy, myocardial infarction, angina, peripheral vascular disease, and ischemic stroke. The eight non-cardiometabolic diseases included were arthritis, anemia, cancer, chronic kidney disease, chronic lung disease, depression, anxiety, and hypothyroidism. Based on the distribution of these chronic conditions in our study participants, we created comparison categories for all types of chronic conditions (1–4, 5–7, ≥8), cardiometabolic (1–2, 3–4, ≥5), and non-cardiometabolic (1–2, 3–4, ≥5) conditions.

### Measurement of physical frailty

The Cardiovascular Health Study (CHS) frailty scale was used to assess the frailty status of study participants, based on five components including unintentional weight loss/shrinking, exhaustion/reduced energy levels, low physical activity, weak grip strength (assessed by a hand dynamometer/grip strength meter), and slow gait speed identified by a 15-ft timed walk [12]. Based on the CHS guidelines, participants with 3 or more of the 5 components were classified as frail, those with 1–2 components were categorized as pre-frail, and participants with none of these components were considered to be non-frail [12].

### Assessment of SRH status

At the time of study enrollment, participant’s SRH status was evaluated with a validated and reliable single-item measure with responses on a 5-point Likert scale which asked: “In general, would you say your health is excellent, very good, good, fair, or poor?” [22]. Due to the relatively small sample sizes in the extreme categories of SRH status, namely those who reported their health as either excellent (*n* = 95) or poor (*n* = 14), we categorized study participants into three groups as either having “excellent/very good”, “good” or “fair/poor” SRH status respectively.

### Baseline participant characteristics

The sociodemographic factors assessed by in-person or telephone interviews included the patient’s age, sex, race/ethnicity, marital status, and highest educational level attained. Clinical measures retrieved from patient medical records and in-person interviews included type of AF, history of AF symptoms in the preceding 4 weeks, receipt of ablation therapy, anticoagulation therapy (directly acting anticoagulants or warfarin), polypharmacy (defined as ≥5 medications) and calculated risk scores including the CHA2DS2-VASc for stroke risk (congestive heart failure, hypertension, age, diabetes, prior stroke/TIA, vascular disease [peripheral arterial disease, previous MI, aortic atheroma] and sex category) [23], HASBLED for 1-year risk of major bleeding (hypertension, abnormal renal and liver function, prior stroke, prior bleeding, labile INR, elderly, drugs or alcohol that increase risk of bleeding) [23], and Charlson Comorbidity index [24]. Psychosocial and geriatric elements derived from comprehensive structured interviews included measures of low social support, cognitive impairment, independent functioning, and self-reports of hearing and visual impairment. Five items of the Medical Outcomes Social Support Survey assessing emotional/informational, tangible, affectionate, and positive social interaction were used to assess social support [25]. The 30-item Montreal Cognitive Assessment Battery (MoCA) was used in assessing participant cognition [26]. The instrumental activities of daily living was utilized in examining level of independence in the following areas: basic communication skills, transportation, meal preparation, shopping, housework, managing medications, and personal finances [27]. Health behaviors such as smoking history and alcohol use were directly reported by participants.

### Statistical analysis

We compared differences in participant’s baseline sociodemographic, clinical, geriatric, and psychosocial characteristics, as well as their health behaviors across the three categories of SRH status (excellent/very good vs. good vs. fair/poor). Continuous variables were summarized either as means and standard deviations with normal distribution patterns, or as medians and interquartile ranges with skewed distribution pattern. We used the non-parametric Wilcoxon rank-sum test to compare continuous variables across the ordered SRH levels. The Cochran–Armitage test for trend was used for between group comparisons for categorical variables.

In examining the association between multimorbidity, physical frailty, and SRH status, ordinal logistic regression models were used to assess both the crude and multi-variable adjusted odds ratios (ORs) and accompanying 95% confidence intervals (CI). Separate regression models were constructed to examine the association between: i) multimorbidity (all comorbid conditions, cardiometabolic, and non-cardiometabolic) and SRH (outcome); ii) physical frailty and SRH (outcome); and iii) multimorbidity and physical frailty (outcome). Potential confounding variables were selected based on clinical judgement and factors associated with multimorbidity, physical frailty, and/or SRH. We systematically examined the impact of a number of potentially confounding variables by sequential adjustment of participant characteristics. First, sociodemographic characteristics were included in the model (age, sex, race/ethnicity, marital status, and highest level of education). Subsequently, clinical variables and health behaviors (symptoms of AF, type of AF, ablation type, alcohol use, and smoking status) were added to the models. Lastly, psychosocial and geriatric measures including low social support, independent functioning, cognitive, hearing, and visual impairment, were adjusted for in the regression model. Physical frailty was also adjusted for as a potential confounder in the regression model assessing the relationship between multimorbidity and SRH, and multimorbidity was adjusted in the model assessing the relationship between frailty and SRH. The proportional odds assumption for the ordinal logistic regression models was sufficiently met when evaluated with the Brant test [28]. Post-hoc analyses were conducted with pairwise comparisons using Bonferroni corrections [29] for multiple testing across the respective multimorbidity categories. A two-sided *p*-value less than 0.05 was considered statistically significant.

## Results

Participants without information on their SRH status (*n* = 9) were excluded from the present analysis, resulting in an analytic sample of 1235 patients diagnosed with AF. The mean age of study participants was 76 years, 49% were women, and 86% were White (Table 1).

**Table 1:** Baseline sociodemographic, clinical, geriatric, and psychosocial characteristics of study participants overall and by self-rated health

Characteristics	Overall Analytic Sample (***n*** = 1235)	Excellent/ Very Good Self-Rated Health (***n*** = 491)	Good Self-Rated Health (***n*** = 540)	Fair/Poor Self-Rated Health (***n*** = 204)	P-value for trend
**Socio-demographic**					
Age (mean, yrs. (sd))	75.5 (7.1)	75.6 (7.1)	75.5 (7.0)	75.0 (7.2)	0.85
Age categories (%)					
65–74 years	50.5	50.1	50.6	51.0	0.73
75–84 years	36.4	36.5	36.1	37.2	
≥ 85 years	13.1	13.4	13.3	11.8	
Women (%)	48.7	45.6	50.4	51.5	0.10
Race/Ethnicity (%)					
White	86.3	92.4	86.8	70.1	**< 0.001**
Non-White	13.7	7.6	13.2	29.9	
Married (%)	56.7	62.3	54.4	49.2	**< 0.01**
Education					
≤ high school	37.5	28.2	40.6	52.3	**< 0.001**
Some college	19.3	17.0	21.4	19.3	
College graduate	43.2	54.8	38.0	28.4	
**Clinical**					
AF Type (%)					
Paroxysmal	66.4	70.6	62.5	66.8	0.18
Persistent	15.1	12.4	16.9	16.6	
Permanent	18.5	17.0	20.6	16.6	
Ablation Therapy (%)	30.7	28.3	31.8	33.3	0.14
Symptoms of AF in past 4 weeks (%)	29.6	25.9	31.2	34.2	**0.02**
Anticoagulation therapy (%)					
DOAC	37.5	36.5	38.5	37.3	0.30
Warfarin	48.2	46.8	49.1	49.0	
None	14.3	16.7	12.4	13.7	
Polypharmacy (≥5 medications) (%)	28.4	22.4	28.5	42.6	**< 0.001**
CHA2DS2-VASc > 2 (%)	89.1	84.1	90.6	97.1	**< 0.001**
HASBLED ≥3 (%)	74.1	69.6	75.9	79.9	**< 0.01**
Charlson comorbidity index, (mean, SD)	6.0 (2.6)	5.5 (2.3)	6.1 (2.5)	7.2 (2.9)	**< 0.001**
Charlson comorbidity index, categories (%)					
1–2	4.9	7.9	3.3	2.0	**< 0.001**
3–4	26.3	31.0	26.1	15.7	
≥ 5	68.8	61.1	70.6	82.4	
**Psychosocial and Geriatric**					
Low social support (%)	26.6	21.0	28.7	34.3	**< 0.001**
Cognitive impairment (%)	35.9	29.6	36.7	49.0	**< 0.001**
Hearing impairment (%)	36.2	33.6	35.2	45.1	**0.01**
Visual impairment (%)	34.2	17.3	38.3	64.2	**< 0.001**
Frailty (%)					
Not Frail	33.4	47.4	28.7	12.2	**< 0.001**
Pre-Frail	52.8	49.2	56.7	51.0	
Frail	13.8	3.3	14.6	36.8	
Independent functioning (IADLs) (Mean, SD)	6.7 (0.8)	6.9 (0.6)	6.7 (0.8)	6.5 (1.1)	**< 0.001**
**Health behaviors**					
Alcohol use (%)	54.9	65.8	51.7	37.4	**< 0.001**
Smoking status (%)					
Never smoker	46.7	52.1	43.7	41.6	**< 0.01**
Former smoker	50.1	45.7	52.9	53.5	
Current Smoker	3.2	2.3	3.4	4.9	

Abbreviations: *DOAC* Direct Oral Anticoagulant, *CHA2DS2-VASc* Stroke risk assessment (Congestive heart failure, Hypertension, Age (≥ 65 = 1 point, ≥ 75 = 2 points), Diabetes, and prior Stroke/TIA (2 points), Vascular disease (peripheral arterial disease, previous MI, aortic atheroma) and female gender); HASBLED: Determines 1-year risk of major bleeding (Hypertension, Abnormal renal and liver function, prior Stroke, prior Bleeding, Labile INR, Elderly, Drugs or alcohol that increase risk of bleeding); IADLs: Instrumental Activities of Daily Living (score ranging from 0 to 7)

### Study participant characteristics according to self-rated health status

Overall, 40% (*n* = 491) of study participants reported their perceived health status as excellent/very good, 44% (*n* = 540) as good, and 16% (*n* = 204) as fair/poor. Based on our trend test analysis, participants who rated their health status as “fair/poor” were more likely to be non-White and unmarried, and to have less than a high school education compared with those who rated their health as either “excellent/very good” or “good” (Table 1). A significantly higher proportion of those who perceived their health as being “fair/poor” reported experiencing AF symptoms in the 4 weeks prior to study enrollment, had poorer comorbidity and bleeding risk scores, and were taking 5 or more medications (polypharmacy) than those who ranked their health as “excellent/very good” or “good” (Table 1).

### Frequency of previously diagnosed multiple chronic conditions and frailty

Almost all study participants (99.5%, *n* = 1229) had at least one previously diagnosed comorbid condition. The most prevalent cardiometabolic conditions were hypertension (90%), dyslipidemia (80%), and heart failure (37%). The most common non-cardiometabolic conditions were arthritis (51%), anemia (31%), and cancer (30%). Participants who rated their health as fair/poor tended to have a higher prevalence of the cardiometabolic and non-cardiometabolic conditions (Table 2).

**Table 2:** Prevalence of previously diagnosed conditions, overall and by self-rated health status: SAGE-AF, 2015–2018 (*n* = 1235)

Comorbidities	Overall Prevalence n (%)	Excellent/Very Good Self-Rated Health (***n*** = 491)	Good Self-Rated Health (***n*** = 540)	Fair/Poor Self-Rated Health (***n*** = 204)	P-value for trend
Cardiometabolic (*n* = 10), %					
Hypertension	1113 (90.1)	87.0	90.5	96.6	**< 0.001**
Dyslipidemia	988 (80.0)	78.6	80.2	82.8	0.21
Congestive heart failure	459 (37.2)	23.6	41.8	57.3	**< 0.001**
Diabetes mellitus	384 (31.1)	20.2	31.5	56.4	**< 0.001**
Valvular heart disease	306 (24.8)	24.0	24.8	26.5	0.51
Cardiomyopathy	268 (21.7)	16.1	22.2	33.8	**< 0.001**
Myocardial infarction	241 (19.5)	16.9	17.8	30.4	**< 0.001**
Angina	195 (15.8)	13.0	15.2	24.0	**< 0.01**
Peripheral vascular disease	177 (14.3)	12.4	14.6	18.1	0.05
Ischemic stroke	121 (9.8)	8.1	10.0	13.2	0.04
Non-Cardiometabolic (*n* = 8), %					
Arthritis	628 (50.8)	45.4	53.0	58.3	**< 0.01**
Anemia	388 (31.4)	25.2	31.7	45.6	**< 0.001**
Cancer	377 (30.5)	31.4	30.6	28.4	0.47
Chronic kidney disease	352 (28.5)	22.0	28.9	43.1	**< 0.001**
Chronic lung disease	314 (25.4)	17.3	25.9	43.6	**< 0.001**
Depression	296 (24.0)	17.5	26.8	31.9	**< 0.001**
Anxiety	287 (23.2)	21.4	24.4	24.5	0.27
Hypothyroidism	272 (22.0)	20.8	22.0	25.0	0.24

Overall, slightly over one-third of study participants had 1–4 (*n* = 441) or 5–7 (*n* = 460) previously diagnosed comorbid conditions respectively, while one-quarter (*n* = 328) had 8 or more chronic conditions. The distribution of cardiometabolic conditions were as follows: 1–2 (*n* = 390), 3–4 (*n* = 514), ≥5 (*n* = 309) conditions. The non-cardiometabolic conditions were distributed as the following: 1–2 (*n* = 480), 3–4 (*n* = 414), and ≥ 5 (*n* = 210) conditions (Table 3).

**Table 3:** “Fair/Poor” vs. “Good” or “Very Good/Excellent” Self-Rated Health with multimorbidity category as an independent variable

Multimorbidity Categories	Prevalence n (%)	Unadjusted OR (95% CI)	Model 1 OR (95% CI)	Model 2 OR (95% CI)	Model 3 OR (95% CI)
All Multimorbidity					
*0	6 (0.5)				
1–4	441 (35.7)	Ref	Ref	Ref	Ref
5–7	460 (37.2)	**1.60 (1.24–2.05)**	**1.50 (1.16–1.94)**	**1.51 (1.11–2.05)**	**1.37 (1.02–1.83)**
8 or more	328 (26.6)	**4.12 (3.11–5.47)**	**3.26 (2.43–4.37)**	**3.24 (2.29–4.59)**	**2.15 (1.53–3.03)**
Cardiometabolic					
*0	22 (1.8)				
1–2	390 (31.6)	Ref	Ref	Ref	Ref
3–4	514 (41.6)	**1.77 (1.37–2.27)**	**1.61 (1.24–2.09)**	**1.72 (1.30–2.29)**	**1.51 (1.12–2.03)**
5 or more	309 (25.0)	**3.48 (2.60–4.66)**	**2.91 (2.15–3.95)**	**2.74 (1.97–3.82)**	**2.21 (1.56–3.14)**
Non-Cardiometabolic					
*0	131 (10.6)				
1–2	480 (38.9)	Ref	Ref	Ref	Ref
3–4	414 (33.5)	**1.74 (1.35–2.23)**	**1.55 (1.20–2.00)**	**1.50 (1.14–1.98)**	1.33 (0.99–1.77)
5 or more	210 (17.0)	**2.99 (2.19–4.09)**	**2.47 (1.60–3.21)**	**2.33 (1.64–3.29)**	**1.54 (1.06–2.22)**

*Participants with 0 comorbidities are not included in the regression models

ORs and 95% Confidence intervals were obtained from ordinal logistic regression models

Model 1: Adjusted for sociodemographic variables: age, sex, race, marital status, and highest level of education

Model 2: Adjusted for variables in model 1, clinical variables (symptoms of AF, type of AF, ablation type) and health behaviors (alcohol use and smoking status)

Model 3: Adjusted for variables in model 2 and psychosocial/geriatric measures (low social support, independent functioning, cognitive impairment, hearing impairment, visual impairment, and frailty status)

Approximately 14% (*n* = 170) of study participants were classified as being frail, slightly more than half as pre-frail (*n* = 659), and approximately one-third were considered to be non-frail (*n* = 413) (Table 2).

### Association between multimorbidity, physical frailty, and SRH

From the fully adjusted ordinal logistic regression models, patients were more likely to report poorer SRH, with increasing number of all comorbidities (OR: 2.15 [95% CI: 1.53–3.03], ≥ 8 vs 1–4; OR: 1.37 [95% CI: 1.02–1.83], 5–7 vs 1–4), cardiometabolic (OR: 2.21 [95% CI: 1.56–3.14], ≥ 5 vs 1–2; OR: 1.51 [95% CI: 1.12–2.03], 3–4 vs 1–2), and non-cardiometabolic (OR: 1.54 [95% CI: 1.06–2.22], ≥ 5 vs 1–2) conditions (Table 3). We found some heterogeneity in the effect estimates from the cardiometabolic versus non-cardiometabolic regression models. The odds ratios from the fully adjusted cardiometabolic regression models were of higher magnitude and remained statistically significant for the respective multimorbidity group comparisons (Table 3). After use of Bonferroni corrections, we observed statistically significant pairwise comparisons for all types of multimorbidity (≥8 vs 1–4 and 5–7) and the cardiometabolic (≥5 vs 1–2) conditions (*p* < 0.01).

In the multivariable ordinal logistic regression model assessing the relationship between frailty categories and SRH status, pre-frailty (OR: 1.73 [95% CI: 1.30–2.30]) and frailty (OR: 6.81 [95% CI: 4.34–10.68]) were significantly associated with worse SRH status (Table 4).

**Table 4:** “Fair/Poor” vs. “Good” or “Very Good/Excellent” Self-Rated Health status with physical frailty status as an independent variable

Frailty Status	Prevalence n (%)	Unadjusted OR (95% CI)	Model 1 OR (95% CI)	Model 2 OR (95% CI)	Model 3 OR (95% CI)
Not Frail	413 (33.4)	Ref	Ref	Ref	Ref
Pre-Frail	652 (52.8)	**2.31 (1.81–2.94)**	**2.17 (1.68–2.80)**	**1.87 (1.41–2.47)**	**1.73 (1.30–2.30)**
Frail	170 (13.8)	**10.75 (7.47–15.46)**	**9.44 (6.42–13.89)**	**7.42 (4.81–11.45)**	**6.81 (4.34–10.68)**

ORs and 95% Confidence intervals were obtained from ordinal logistic regression models

Model 1: Adjusted for sociodemographic variables: age, sex, race, marital status, highest level of education

Model 2: Adjusted for variables in model 1, clinical variables (symptoms of AF, type of AF, ablation type, and multimorbidity) and health behaviors (alcohol use and smoking status)

Model 3: Adjusted for variables in model 2 and psychosocial/geriatric measures (low social support, independent functioning, cognitive impairment, hearing impairment, and visual impairment)

Participants with a higher burden of all types of comorbid conditions were more likely to be frail (OR: 1.65 [1.20–2.28], ≥ 8 vs 1–4) as were those with more prevalent cardiometabolic conditions (OR:1.58 [1.12–2.21], ≥ 5 versus 1–2) (Table 5).

**Table 5:** “Frail” vs. “Pre-Frail” or “Not Frail” status with multimorbidity category as an independent variable

Multimorbidity Categories	Prevalence n (%)	Unadjusted OR (95% CI)	Model 1 OR (95% CI)	Model 2 OR (95% CI)	Model 3 OR (95% CI)
All Multimorbidity					
*0	6 (0.5)				
1–4	441 (35.7)	Ref	Ref	Ref	Ref
5–7	460 (37.2)	**1.48 (1.14–1.91)**	**1.36 (1.04–1.77)**	**1.43 (1.08–1.91)**	**1.38 (1.03–1.83)**
8 or more	328 (26.6)	**1.99 (1.51–2.62)**	**1.60 (1.20–2.13)**	**1.72 (1.25–2.37)**	**1.65 (1.20–2.28)**
Cardiometabolic					
*0	22 (1.8)				
1–2	390 (31.6)	Ref	Ref	Ref	Ref
3–4	514 (41.6)	**1.68 (1.30–2.17)**	**1.46 (1.12–1.91)**	**1.38 (1.03–1.84)**	1.32 (0.99–1.77)
5 or more	309 (25.0)	**2.09 (1.57–2.79)**	**1.68 (1.24–2.28)**	**1.66 (1.19–2.31)**	**1.58 (1.12–2.21)**
Non-Cardiometabolic					
*0	131 (10.6)				
1–2	480 (38.9)	Ref	Ref	Ref	Ref
3–4	414 (33.5)	1.26 (0.98–1.63)	1.16 (0.89–1.52)	1.28 (0.96–1.70)	1.25 (0.94–1.66)
5 or more	210 (17.0)	**1.41 (1.04–1.91)**	1.26 (0.92–1.73)	1.34 (0.95–1.89)	1.28 (0.90–1.81)

*Participants with 0 comorbidities are not included in the regression models

ORs and 95% Confidence intervals were obtained from ordinal logistic regression models

Model 1: Adjusted for sociodemographic variables: age, sex, race, marital status, highest level of education

Model 2: Adjusted for variables in model 1, clinical variables (symptoms of AF, type of AF, ablation type) and health behaviors (alcohol use and smoking status)

Model 3: Adjusted for variables in model 2 and psychosocial/geriatric measures (low social support, independent functioning, cognitive impairment, hearing impairment, and visual impairment)

## Discussion

In this contemporary cohort of patients with AF, we observed a high prevalence of cardiometabolic and non-cardiometabolic multimorbidity. One in six patients perceived their overall health status as being fair/poor. More than one-half of study participants met the criteria for being pre-frail, whereas one in seven were classified as being frail. In the fully adjusted regression models, we showed that multimorbidity was strongly associated with worse SRH status and physical frailty; and participants who were pre-frail or frail were more likely to report fair/poor SRH status.

### Prevalence of cardiometabolic and non-cardiometabolic conditions

Consistent with reports from prior studies among patients with AF [8, 30, 31], hypertension, dyslipidemia, and congestive heart failure were the most prevalent cardiometabolic comorbidities diagnosed in our study participants. The pathophysiologic basis and clinical interactions between AF and coexisting cardiometabolic conditions are well established in the literature [32, 33], however, living and coping with the burden of these diseases especially with regards to their synergistic effect on one’s overall well-being and functionality, may be of utmost concern to patients and their caregivers.

Overall, non-cardiometabolic conditions had a lower prevalence compared with the cardiometabolic diseases. Very few studies have examined the prevalence of non-cardiometabolic comorbidities in older patients with AF [34, 35]. In the present study, arthritis (51%), anemia (31%), and cancer (30%) were most common, which was inconsistent with the few existing studies that identified renal failure (10–22%) and chronic lung disease (10–26%) as more commonly occurring non-cardiometabolic conditions in these patients [34, 35]. A potential reason for the lack of consistency across studies in the prevalence of non-cardiometabolic comorbidities may be due to the variability in selected conditions, and whether these conditions are self-reported or obtained from medical records [36]. There is currently no consensus or internationally accepted standard on how the co-occurrence of diseases should be ascertained [37, 38]. Since cardiometabolic conditions occur more frequently in patients with underlying cardiovascular disease [8, 30, 31], it is more likely that they are similar across studies. However, there needs to be a more standardized approach to assessing both cardiometabolic and non-cardiometabolic comorbid conditions to ensure better reliability and consistency across studies. Furthermore, in the context of more integrated approaches to AF management that emphasize the management of accompanying comorbidities to improve AF-related outcomes, healthcare providers should consider integration of information about the patient’s burden of chronic comorbid conditions when considering interventions focused on SRH status, such as ablation or anti-arrhythmic medication administration [4, 5].

### Prevalence of physical frailty

Overall, 14% of our study participants met the criteria of being frail while slightly over one-half were considered to be pre-frail, which is in keeping with prior reports from studies among elderly patients with AF in the outpatient setting [39, 40]. In contrast, prior research among hospitalized elderly patients with AF has shown a greater prevalence of frailty ranging from 35 to 80% [41, 42]. In addition, AF has been identified as a potential marker of frailty in older adults [43]. In a study among 23,174 hospitalized patients, AF was found to be more prevalent among older persons and associated with greater comorbidity, longer in-hospital stay, and worse metabolic profile, suggesting that AF could be a possible indicator of frailty in the elderly [43]. Despite the high burden of multimorbidity among our study participants, our findings of a higher prevalence of pre-frailty as opposed to frailty is consistent with reports that a majority of older persons with multimorbidity are not phenotypically frail, whereas most of those with frailty have multimorbidity [44], further emphasizing that multimorbidity and frailty are interrelated but distinct concepts.

Our results have clinical implications in outpatient management, reinforcing the need for a more holistic and patient-centered approach to chronic disease management in those responsible for managing patients with AF, including primary care, cardiology, and electrophysiology healthcare providers, by incorporating assessments of frailty among elderly patients, especially among those with multiple chronic conditions. Currently, there is no gold standard for assessing physical frailty in clinical practice [45]. Most tools assess between 1 and 5 domains of the components of frailty: weight loss/shrinking, exhaustion, low physical activity, weak grip strength, and slow gait speed [12]. Multidimensional measures of frailty that incorporate all 5 domains may not be easy to utilize in the clinic setting given time constraints and competing demands of busy clinic encounters [46]. Hence, a more simplified and validated method that adequately captures the key components of physical frailty, such as the short physical performance battery, may be more ideal for use in the clinic setting [47]. Routine frailty assessment during clinical encounters may help identify patients who are pre-frail and have a greater predisposition to declining health status. This provides an opportunity to institute timely interventions targeting psychosocial or geriatric elements such as social isolation, unexplained weight loss, and reduced mobility, while optimally managing other coexisting diseases.

### Participant perception of their overall well-being

A person’s unique experience and perception of their overall wellbeing can be adequately captured by SRH which also reflects one’s ability to thrive in their current environment [48, 49]. We found that participants who perceived their health as fair/poor were more likely to belong to ethnic minority groups, had low social support networks, were less educated, and reported lower income in comparison with those who ranked their health as either “excellent/very good” or “good”. Our results emphasize that one’s social environment and support networks influences how they perceive their well-being [49, 50]. In addition, patients who reported experiencing symptoms of AF in the 4 weeks prior to study enrollment, had poorer stroke and bleeding risk scores, and were taking 5 or more medications, were more likely to perceive their overall health status as being fair/poor. These findings suggest the need for healthcare providers to be increasingly aware of patients’ individual needs and the perception of their well-being, since this may very much influence their illness experience and recovery.

### Association between multimorbidity, physical frailty and SRH

The strong dose-response relationship that we observed between SRH, multimorbidity, and physical frailty may be based on bidirectional mechanisms rather than unidirectional causal pathways, with the presence of one fostering the development of the other. For example, in the presence of multiple chronic illnesses, an individual who perceives their overall health status as being fair/poor may have decreased resilience, less optimism regarding their health, and a dysfunctional internal regulatory mechanism, leading to an increased susceptibility to the onset of more chronic diseases and frailty [51]. Indeed, in a recent systematic review, optimism and resilience were linked with a reduced risk of cardiovascular disease and all-cause mortality [52]. Internal coping mechanisms have been shown to be linked with better cardiovascular health which may in turn reduce the burden of multimorbidity [53].

Furthermore, we observed that cardiometabolic conditions had stronger associations with poorer perception of one’s overall health status, and frailty. A possible reason for this stronger relationship may be that with more frequent follow-up visits for their underlying cardiovascular disease, patients may be increasingly more aware of their coexisting cardiometabolic diseases, and this heightened awareness may overwhelmingly impact their general functional status and quality of life. Measures that directly assess how individual comorbid conditions could impact patients’ quality of life may provide a better understanding of the impact of each coexisting disease on patients’ overall their well-being [54]. In a recent study among elderly adults with AF, cardiometabolic multimorbidity was associated with worse clinical outcomes including a greater risk of stroke, severe bleeding, and heart failure [55]. Future longitudinal studies should explore potential mechanisms by which multimorbidity, physical frailty, and SRH may be interrelated and their impact on important clinical outcomes, to ensure a more holistic approach in the delivery of patient-centered care.

### Study strengths and limitations

Our study has several important strengths. First, we utilized contemporary data from a multi-center cohort of older men and women managed for AF with detailed clinical, psychosocial, and geriatric characteristics. Second, the measures of physical frailty and SRH employed in this study are well established and widely accepted measures, enhancing the validity and reproducibility of our results. In addition, we ascertained the chronic comorbid conditions from medical records which reduces the likelihood of misclassification bias with over or underestimation from patient self-reports. Our study findings should be interpreted in light of certain limitations, however. Given our cross-sectional study design, we were unable to establish temporality and causality between multimorbidity, physical frailty, and SRH. Furthermore, the comorbid conditions included in the present study are inexhaustive as other important age-related conditions such as sarcopenia (progressive loss of skeletal muscle mass and strength) which may impact physical frailty [56] were not assessed. Also, our findings have limited generalizability to ethnic minority groups since most of our study participants were White, and culture plays an important role in people’s perception of their well-being. Future studies should be conducted in more ethnically diverse patient populations with AF.

## Conclusions

Cardiometabolic and non-cardiometabolic comorbid conditions occur very commonly among patients with AF. The presence of multimorbidity and physical frailty was associated with one’s perception of their overall well-being, with a stronger association observed with increasing number of cardiometabolic conditions and frailty. Our findings suggest that cardiometabolic conditions may exert a more significant effect on SRH than non-cardiometabolic conditions in patients with AF. These findings have important implications in this contemporary era focusing on a more holistic approach to the management of comorbidities among patients with various coexisting chronic conditions, further emphasizing the need to take into consideration patients’ perception of their well-being when making therapeutic decisions especially in the setting of multimorbidity and/or physical frailty.

## Data Availability

The datasets during and analyzed during the current study available from the corresponding author on reasonable request.

## Abbreviations

AF: Atrial fibrillation
CHA2DS2-VASc: Congestive heart failure, Hypertension, Age, Diabetes, Prior Stroke/TIA, Vascular disease, Sex Category
CHS: Cardiovascular Health Study
DOAC: Direct Oral Anticoagulants
HASBLED: Hypertension, Abnormal renal and liver function, prior Stroke, prior Bleeding, Labile INR, Elderly, Drugs or alcohol that increase risk of bleeding
IADLs: Instrumental Activities of Daily Living
INR: International Normalized Ratio
MOCA: Montreal Cognitive Assessment Battery
SAGE-AF: Systematic Assessment of Geriatric Elements in AF
SRH: Self-rated health
US-DHHS: US Department of Health and Human Services
